# Evaluation of the quality of life among transgender men before and after gender reassignment surgery: a survey from Iran

**DOI:** 10.1186/s13034-024-00794-0

**Published:** 2024-08-10

**Authors:** Elham Rahimpour, Elham Askary, Shaghayegh Moradi Alamdarloo, Saeed Alborzi, Tahereh Poordast

**Affiliations:** 1 Department of Obstetrics and Gynecology, School of Medicine, Maternal-Fetal Medicine Research Center, Shiraz University of Medical Sciences, Shiraz, Iran; 2 Department of Obstetrics and Gynecology, School of Medicine, Laparoscopy Research Center, Shiraz University of Medical Sciences, Shiraz, Iran; 3 Department of Obstetrics and Gynecology, School of Medicine, Infertility Research Center, Shiraz University of Medical Sciences, Shiraz, Iran; 4 Present Address: Obstetrics and Gynecology Office, Shahid Faghihi Hospital, Zand Avenue, Shiraz, 7134844119 Iran

**Keywords:** Gender reassignment surgery, Gender dysphoria, Quality of life

## Abstract

**Background:**

Gender dysphoria, characterized by a misalignment between one’s gender identity and assigned sex, propels individuals towards medical interventions like gender reassignment surgery (GRS) to harmonize their bodies with their gender. This process aims to enhance overall quality of life (QoL), functioning, and body image. Recognizing the importance of cultivating a positive body image for transgender individuals navigating societal norms, this narrative highlights the ongoing debate surrounding QoL post-GRS. In response, our study is outlined, aiming to scrutinize QoL and self-image among transgender men post-GRS, offering valuable insights into societal perceptions and psychological well-being in this context.

**Method:**

This cross-sectional survey focused on transgender men aged 15 to 35 who underwent gender reassignment surgery (GRS) in 2018–2022 in Shiraz, Iran. Participants, after passing psychiatric evaluations, completed World Health Organization Quality of Life (WHOQOL-100) questionnaires pre- and at least one-year post-surgery. The scores of the Brief-WHOQOL questionnaire were evaluated in four domains of physical health, psychological health, social relationships, and environmental health.

**Results:**

A total of 60 individual who underwent GRS completed our questionnaire. The average age of the patients was 24.1 ± 3.8 years. Following GRS, the most increase was observed in the psychological factor (by 25.6%). The increase in score was statistically significant in all subgroups (*P* < 0.001) after operation. Urban living location had a significant association with higher increase in physical health (*P* < 0.010), psychological health (*P* = 0.005), and environmental health (*P* = 0.012) after GRS. In regards to physical health, the low socioeconomic group had a significantly less physical score improvement in QoL compared to the moderate group (*P* = 0.024) following GRS. In regards to environmental health, the high socioeconomic groups had significantly higher improvement in QoL compared to the low (*P* = 0.006) and moderate (*P* < 0.001) group after operation.

**Conclusion:**

The results demonstrate that GRS brings about improvements across all aspects of QoL. However, this enhancement is less pronounced among patients hailing from low socioeconomic backgrounds and rural areas.

## Background

Gender dysphoria is a is described as a persistent and distressing misalignment between gender identity and the sex assigned at birth [1]. To alleviate the physical incongruence and distress associated with gender dysphoria, there is often a strong desire for medical and surgical interventions to align the body more closely with one’s experienced gender [2]. The prevalence of gender dysphoria seems to be increasing globally [3–5].

Transgender individuals often experience a sense of being trapped in the wrong gendered body and typically seek hormonal and surgical reassignment to align with their identified gender. Many desire the removal of the uterus and ovaries promptly to alleviate the stress of menstrual occurrences and reduce estrogen production, facilitating the effectiveness of exogenous androgen therapy [6].

Quality of life (QoL) is characterized as an “individuals’ perceptions of their position in life concerning their goals, expectations, standards, and concerns in the context of the culture and value systems in which they live.” This encompasses a broad concept influenced by factors such as physical health, psychological well-being, level of independence, social relationships, personal beliefs, and the connection to significant aspects of their environment. The intricate interplay of these elements contributes to the overall understanding of an individual’s quality of life. [7–10]

Gender reassignment surgery (GRS) is widely acknowledged for enabling transgender individuals to embrace their identified gender fully, marking it as the most effective treatment option [11]. Both GRS and hormonal treatment (HT) have demonstrated enhancements in quality of life (QoL), overall functioning, and body image perception. Despite variations in hormonal treatment dosages in some studies and the absence of standardized assessments for hormonal status, positive effects have been observed. Additionally, some experts suggest that GRS and HT might contribute to a reduction in the risk of suicidal attempts among transgender individuals [11–14]. 

Transgender individuals should have the opportunity to cultivate a positive body image [15]. For transgender people, body image serves as a means of self-expression and enables them to navigate their transgender identity in a world that often perceives gender in binary terms. This dynamic gives rise to a complex interplay of desire, authenticity, and the need to avoid societal stigma. Achieving a positive body image is crucial for trans people as it empowers them to assert their identity and cope with the challenges posed by societal norms and expectations. [16]

There exists a lack of consensus in the field regarding QoL, particularly post-gender reassignment surgery [17]. Some earlier studies indicate that transgender individuals exhibit lower QoL compared to the general population [17–19], while others report no significant differences in QoL or psychological functioning between transgender individuals and the general population [14, 20–22]. Poor sexual life quality post-surgery can negatively impact psychological well-being, causing considerable distress [23]. Thus, this study seeks to assess the quality of life and self-image among transgender men following gender reassignment surgery (GRS), aiming to contribute valuable insights to the ongoing discourse in this area.

Homosexuality is banned in Iran, but gender reassignment (GR) has been religiously permitted since 1987 after the Iranian revolution. Iran stands as the sole Islamic nation endorsing and financially supporting GRS procedures. As per the Family Protection Law since 2012, any individual in Iran may submit their request for gender matching to the family court there. According to Ahmadzadeh’s study between 2002 and 2009, the annual application rate for transgender women was approximately 1 in 145,000, and for transgender men was around 1 in 136,000. In 2022, this figure was accompanied by a two-fold increase in requests by transgender men compared to transgender women, highlighting the increasing prevalence of gender reassignment in Iran. [24, 25].

This study marks a significant milestone as it is the first of its kind conducted among Iranians, shedding light on the quality of life and self-image among transgender men post-GRS. It is noteworthy to mention that this research highlights the cultural and religious context that is still evolving, with acceptance by families being a recent development and societal integration still in progress. The strengths of this study lie in its comprehensive examination of various aspects related to transgender healthcare within an Iranian cultural framework, offering valuable insights into the experiences of transgender individuals in a society where such topics are relatively emerging.

## Material and method

In this cross-sectional survey study, we included transgender men participants who underwent GRS between 2018 and 2022 (4 years) at Shahid Faghihi, Zeinabiyyeh, Peyvand, Ali-Asghar and Madar-kodak Hospitals, the five major hospitals affiliated with Shiraz University of Medical Sciences in Shiraz, Iran. GRS was performed among the participants through the laparoscopic hysterectomy and bilateral salpingo -oophrectomy method. All participants underwent a psychiatric evaluation to ensure the absence of severe psychiatric disorders, excluding any lifetime history of organic mental disorders, mental retardation, psychotic disorders, bipolar disorders, substance abuse, and severe Axis II psychopathology (cluster A personality disorder, antisocial personality disorder, and borderline personality disorder) according to the DSM-V [26].

Before undergoing surgery to remove the uterus and ovaries, individuals had already undergone mastectomy and were living with a man. A new birth certificate will be issued to them immediately following the hysterectomy. Patients often avoided discussing surgery related to external genitalia, despite it being a crucial aspect of the GRS. This could be due to the fact that, talking about genitalia in Iran is culturally sensitive, leading to reliance on non-verbal cues for sexual communication and seeking satisfaction in sexual roles.

The questionnaires were administered twice during the study period, once before the surgery and once at least one year after undergoing GRS. patients were contacted through a telephone survey, and after explaining the aim and details of the study, they provided verbal informed consent. All patients agreed to the participation in the study, sharing and publishing their information and questionnaires.

We utilized the Persian version of the WHOQOL-BREF questionnaire for the purpose of our study [27, 28]. The WHOQOL-BREF is a 26-item tool with four domains: physical health (7 items), psychological health (6 items), social relationships (3 items), and environmental health (8 items), including QOL and general health items. Each item is rated on a five-point ordinal scale (1 to 5), and scores are linearly transformed to a 0–100 scale [29, 30]. In the physical health domain, items assess mobility, daily activities, functional capacity, energy, pain, and sleep. The psychological domain covers self-image, negative thoughts, positive attitudes, self-esteem, mentality, learning ability, memory concentration, religion, and mental status. Social relationships involve personal relationships, social support, and sex life. The environmental health domain addresses financial resources, safety, health and social services, living physical environment, opportunities for skills and knowledge, recreation, general environment (noise, air pollution, etc.), and transportation. Cultural differences do not influence the importance of the domains. The scores ranged from 1 to 5 for each question, and ranged from 7 to 35 for physical health, 6 to 30 for psychological health, 3 to 15 for social relationships, and 8 to 40 for and environmental health (8 items).

The questionnaire has been translated and validated in Persian language [27]. Subjects rate each item on a Likert scale ranging from 1 to 4 or 1 to 5 [31]. We also added two extra questions of “Do you have any issues in your sexual life” and “To what extent are your sexual needs met?”. The answers consisted of “not at all/very poor/ very dissatisfied/ never as 0, not much/poor/ dissatisfied/seldom as 1, moderately/neither poor nor good/ neither satisfied nor dissatisfied/ quit often as 2, a great deal/good/ satisfied / very often as 3, and completely/very good/ very satisfied/ an extreme amount/ always as 4. Score calculation was performed according to the WHOQOL manual [28].

All analyses were performed using the Statistical Package for Social Science (SPSS v.27.0 software). Distribution was summarized through means and standard deviations (mean ± SD) or median and interquartile range (IQR). Descriptive statistics are reported as frequency and percentage (%). Wilcoxon signed test was applied to evaluate differences between the responses before and after surgery. Statistical significance was accepted at the two-tailed *P* < 0.05 significance level.

## Results

A total of 60 transgender men individual who underwent GRS completed our questionnaire. The average age of the patients before operation was 24.1 (SD: 3.8; range: 13–31). Table 1 demonstrated the demographic features of the patients in our study. None of our participants were married.

**Table 1:** Demographic features of participants undergoing gender reassessment surgery

Variable		Value; *N* = 60
Age (years); mean ± standard deviation		24.1 ± 3.8
Marital status; n (%)	Single	53 (88.3)
	Divorced	7 (11.7)
Education level; n (%)	Under Diploma	1 (1.7)
	Diploma	33 (55.0)
	Masters and above	26 (43.3%)
Residence; n (%)	Urban	37 (61.7)
	Rural	33 (38.3)
Financial status; n (%)	Low	12 (20.0)
	Moderate	30 (50.0)
	High	18 (30.0)

The participants filled out the Brief-WHOQOL questionnaire at two phases of before operation and after operation. The median interval between the filling of questionnaire was 3 years. The descriptive frequency of the responses in our study is demonstrated in Table 2.

**Table 2:** Frequency of responses of patients undergoing gender reassessment surgery based on quality of life before and after surgery

WHO Code	Questions	Preoperative score					Post-operative score					Change**	
		1	2	3	4	5	1	2	3	4	5	Average (%)	*P*-value
G1	How would you rate your quality of life?	0 (0)	11 (18.3)	46 (76.7)	3 (5.0)	0 (0)	0 (0)	1 (1.7)	20 (33.3)	28 (46.7)	11 (18.3)	33.1	< 0.001
G4	How satisfied are you with your health?	0 (0)	1 (1.7)	43 (71.7)	16 (26.7)	0 (0)	0 (0)	14 (23.3)	27 (45.0)	19 (31.7)	0 (0)	25.6	< 0.001
Ph; F1.4	To what extent do you feel that (physical) pain prevents you from doing what you need to do? *	10 (16.7)	38 (63.3)	11 (18.3)	1 (1.7)	0 (0)	22 (36.7)	28 (46.7)	10 (16.7)	0 (0)	0 (0)	−12.2	0.016
Ph; F11.3	How much do you need any medical treatment to function in your daily life? *	8 (13.3)	43 (71.7)	19 (15.0)	0 (0)	0 (0)	22 (36.7)	31 (51.7)	7 (11.7)	0 (0)	0 (0)	−13.2	0.008
Psy: F4.1	How much do you enjoy life?	2 (3.3)	32 (53.3)	26 (43.3)	0 (0)	0 (0)	0 (0)	2 (3.3)	35 (58.3)	21 (35.0)	2 (3.3)	41.0	< 0.001
Psy: F24.2	To what extent do you feel your life to be meaningful?	1 (1.7)	37 (61.7)	22 (36.7)	0 (0)	0 (0)	0 (0)	0 (0)	42 (70.0)	17 (28.3)	1 (1.7)	41.1	< 0.001
Psy: F5.3	How well are you able to concentrate?	1 (1.7)	20 (33.3)	39 (65.0)	0 (0)	0 (0)	0 (0)	7 (11.7)	31 (51.7)	22 (26.7)	0 (0)	23.4	< 0.001
En; F16.1	How safe do you feel in your daily life?	1 (1.7)	9 (15.0)	50 (83.3)	0 (0)	0 (0)	0 (0)	8 (13.3)	34 (56.7)	17 (28.3)	1 (1.7)	13.0	< 0.001
En; F22.1	How healthy is your physical environment?	0 (0)	12 (20.0)	47 (78.3)	1 (1.7)	0 (0)	0 (0)	6 (10.0)	33 (55.0)	17 (28.3)	4 (6.7)	17.8	< 0.001
Ph; F2.1	Do you have enough energy for everyday life?	0 (0)	30 (50.0)	30 (50.0)	0 (0)	0 (0)	0 (0)	2 (3.3)	40 (66.7)	14 (23.3)	4 (6.7)	33.3	< 0.001
Psy: F7.1	Are you able to accept your bodily appearance?	28 (46.7)	30 (50.0)	2 (3.3)	0 (0)	0 (0)	0 (0)	9 (15.0)	27 (45.0)	18 (30.0)	6 (10.0)	113.8	< 0.001
En; F18.1	Have you enough money to meet your needs?	0 (0)	14 (23.3)	43 (71.7)	2 (3.3)	1 (1.7)	1 (1.7)	12 (20.0)	38 (63.3)	7 (11.7)	2 (3.3)	4.12	0.090
En; F20.1	How available to you is the information that you need in your day-to-day life?	0 (0)	15 (25.0)	42 (70.0)	3 (5.0)	0 (0)	2 (3.3)	13 (21.7)	34 (56.7)	10 (16.7)	1 (1.7)	4.17	0.194
En; F21.1	To what extent do you have the opportunity for leisure activities?	0 (0)	26 (43.3)	34 (56.7)	0 (0)	0 (0)	0 (0)	17 (28.3)	30 (50.0)	12 (20.0)	1 (1.7)	14.9	< 0.001
Ph; F9.1	How well are you able to get around?	0 (0)	5 (8.3)	41 (68.3)	14 (23.3)	0 (0)	0 (0)	1 (1.7)	9 (15.0)	35 (58.3)	15 (25.0)	29.1	< 0.001
Ph; F3.3	How satisfied are you with your sleep?	0 (0)	19 (31.7)	40 (66.7)	1 (1.7)	0 (0)	0 (0)	11 (18.6)	11 (18.6)	27 (45.8)	10 (16.9)	31.5	< 0.001
Ph; F10.3	How satisfied are you with your ability to perform your daily living activities?	1 (1.7)	8 (13.3)	48 (80.0)	3 (5.0)	0 (0)	0 (0)	5 (8.3)	19 (31.7)	31 (51.7)	5 (8.3)	24.8	< 0.001
Ph; F12.4	How satisfied are you with your capacity for work?	0 (0)	12 (20.0)	46 (76.7)	2 (3.3)	0 (0)	0 (0)	4 (6.7)	0 (0)	31 (51.7)	25 (41.7)	51.2	< 0.001
Psy: F6.3	How satisfied are you with yourself?	0 (0)	2 (23.3)	42 (70.0)	4 (6.7)	0 (0)	0 (0)	0 (0)	15 (25.0)	37 (61.7)	8 (13.3)	37.1	< 0.001
Sc: F13.3	How satisfied are you with your personal relationships?	2 (3.3)	27 (45.0)	31 (51.7)	0 (0)	0 (0)	0 (0)	11 (18.3)	29 (48.3)	19 (31.7)	1 (1.7)	27.5	< 0.001
Sc: F15.3	How satisfied are you with your sex life?	11 (18.3)	38 (63.3)	11 (18.3)	0 (0)	0 (0)	6 (10.0)	15 (25.0)	23 (38.3)	14 (23.3)	2 (3.3)	46.2	< 0.001
Sc: F14.4	How satisfied are you with the support you get from your friends?	0 (0)	2 (45.0)	29 (48.3)	4 (6.7)	0 (0)	4 (6.7)	24 (40.0)	23 (38.3)	6 (10.0)	3 (5.0)	1.9	0.655
En; F17.3	How satisfied are you with the conditions of your living place?	1 (1.7)	13 (21.7)	34 (56.7)	12 (20.0)	0 (0)	0 (0)	11 (18.3)	22 (36.7)	21 (35.0)	6 (10.0)	14.1	< 0.001
En; F19.3	How satisfied are you with your access to health services?	0 (0)	6 (10.0)	36 (60.0)	18 (30.0)	0 (0)	0 (0)	4 (6.7)	17 (28.3)	34 (56.7)	5 (8.3)	14.6	< 0.001
En; F23.3	How satisfied are you with your transport?	0 (0)	5 (8.3)	32 (53.3)	23 (38.3)	0 (0)	0 (0)	2 (3.3)	0 (0)	29 (48.3)	29 (48.3)	33.8	< 0.001
Psy: F8.1	How often do you have negative feelings such as blue mood, despair, anxiety, depression? *	0 (0)	0 (0)	26 (43.3)	31 (51.7)	3 (5.0)	2 (3.3)	21 (35.0)	21 (35.0)	15 (25.0)	1 (1.7)	−20.7	< 0.001
Extra 1	Do you have any issues in your sexual life? *	0 (0)	0 (0)	6 (10.0)	40 (66.7)	14 (23.3)	11 (18.3)	16 (26.7)	21 (35.0)	7 (11.7)	5 (8.3)	−35.9	< 0.001
Extra 2	To what extent are your sexual needs met?	19 (31.7)	37 (61.7)	4 (6.7)	0 (0)	0 (0)	10 (17.2)	15 (25.9)	24 (41.4)	8 (13.8)	1 (1.7)	41.9	< 0.001

* Questions have reverse scoring

** Change is assessed based on mean difference compared to the pre-operative scores, and analyzed based on the Wilcoxon signed test

Values are presented as frequency (percentage (%))

En: Environmental; Ph: Physical; Psy: Psychological; Sc: Social

As shown in Table 2, and accounting for reverse scoring in several questions (F1.4, F11.3, F8.1, and Extra 1), the responses demonstrated overall improvement in all questions. The increase was significant in all questions following GRS (*P* < 0.05) except three questions: “Have you enough money to meet your needs?”, “How available to you is the information that you need in your day-to-day life?”, and “How satisfied are you with the support you get from your friends?”. The highest improvement was in the question “Are you able to accept your bodily appearance?” by 113%.

**Fig. 1:**
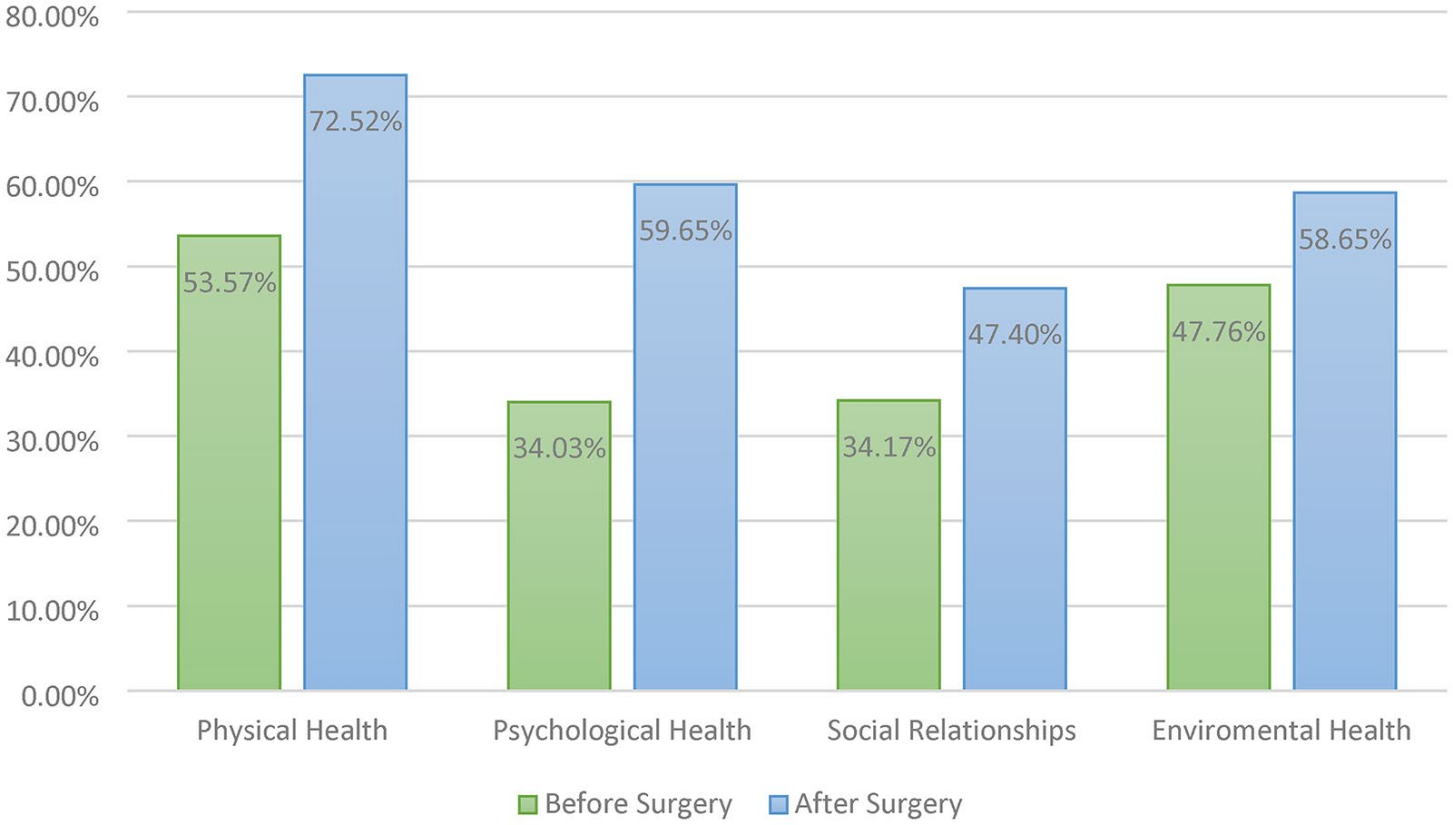
Quality of life subsection score before and after surgery

The scores were evaluated in four domains of physical health, psychological health, social relationships, and environmental health. Figure 1 demonstrates the average score of the participants before and after surgery. The most increase was observed in the psychological factor (by 25.6%), followed by physical health (19.1%), social relationships (13.2%), and environmental health (10.9%). The increase in score was statistically significant in all subgroups (*P* < 0.001).

When evaluating the amount of change in the subgroups, based on the participants demographic features, we observed no significant association with age, marital status, or educational level. Living location had a significant association with physical health (*P* < 0.010), psychological health (*P* = 0.005), and environmental health (*P* = 0.012), but not social relationships (*P* = 0.088), with urban residents demonstrating higher level of change. The socioeconomic status of the participants also had a significant association with physical health (*P* = 0.023) and environmental health (*P* < 0.001), but not psychological health (*P* = 0.596) or social relationships (*P* = 0.684). Based on the post-hoc test results, in regards to physical health, the low socioeconomic group had a significant lower physical score compared to the moderate group (*P* = 0.024), however, there was no significant difference between the moderate and high group (*P* = 1.000) or the low and high group (*P* = 0.070). In regards to environmental health, the high socioeconomic groups had significantly higher improvement compared to the low (*P* = 0.006) and moderate (*P* < 0.001) group.

## Discussion

A total of 60 transgender men who underwent GRS completed our questionnaire. The average age of the patients before operation was 24.1, which was lower to similar studies [20, 32]. The majority of participants were educated, which is in accordance to a previous study by Cardoso da Silva et al. [32]. Most participants resided in urban areas (61.7%) and had a moderate (50%) or high (30%) socioeconomic status. Our results indicated an increase in QoL after GRS, which is line with previous reports [32, 33]. Dhiordan et al. conducted a pre-post survey assessing GRS impact on transgender women in Brazil. Their findings revealed improvements in the psychological social relationships of the WHOQoL-BREF after stereotactic radiosurgery when comparing post-surgery results to pre-surgery evaluations [32]. We observed that the QoL significantly increased, both in overall scores and also in subgroups of physical, psychological, social, and environmental health. These changes were unrelated to patients age, marital status, and education, and more influenced by their socioeconomic status and living location. This demonstrates the importance of the environment and living situation and culture which can influence the individuals’ beliefs and QoL. The patients with a low socioeconomic status demonstrated the lowest change in their physical and environmental health factors. On the other hand, patients with high socioeconomic status demonstrated significant improvement in their environmental health. Also, urban living residents compared to rural residents showed significantly higher improvement in physical, psychological, and environmental health factors. Our study is the first of its kind conducted in Iran, offering a groundbreaking exploration into the quality of life and self-image of transgender men post-GRS within the Iranian context. By comprehensively examining various aspects of their experiences, this study fills a crucial gap in existing research and provides valuable insights into the challenges and successes faced by transgender individuals in Iran. Additionally, its thorough investigation contributes to the advancement of knowledge and understanding in both academic and clinical settings, paving the way for further research and improved support for transgender individuals in Iran and beyond.

In a more detailed evaluation of the responses, the increase in QoL was significant in all questions except three questions: “Have you enough money to meet your needs?”, “How available to you is the information that you need in your day-to-day life?”, and “How satisfied are you with the support you get from your friends?”. These questions represent the socioeconomic status, availability of information, and social support of the participants, respectively. The observable traits of transgender individuals, such as their voice and facial features, along with the behaviors of their friends and family, plays a crucial role in their post-surgery interactions within the community. Transgender women were identified to experience greater limitations and challenges in this regard [34]. Factors like disapproval from family and the community may expose transgender individuals to vulnerability, gradually influencing their QoL and potentially contributing to the onset of depression [35]. Rezaei et al. demonstrated that aspects such as family function, emotional fusion, behavior control, and emotional responsiveness can play a crucial role in facilitating the acceptance of their new sexual role among transgender individuals [36].

Engaging in a range of social activities due to gender reassignment has been observed to enhance the sociability and activity levels of transgender individuals, fostering stronger social connections and helping them overcome social isolation. This enhancement in social relationships has the potential to elevate their overall QoL [37, 38].

A notable aspect of focus in this study is sexual activity, which showed improvement after GRS through the two additional questions we provided. This finding has also been supported in previous studies [32]. One potential explanation for this observation could be linked to a heightened sense of personal fulfillment post-surgery and an enhanced acceptance of one’s body. This is also evident in our study, which the highest improvement was in the question “Are you able to accept your bodily appearance?” by 113%. Bartolucci et al. [23] asserted that GRS serves as a cornerstone for individuals with gender dysphoria, not only addressing their gender dysphoria but also leading to an enhancement in sexual satisfaction.

This study has various limitations, notably the short-term evaluation period post-GRS, the varying recovery times of patients after surgery, and the diverse levels of QoL among individuals. Consequently, we underscore the importance of conducting additional follow-up trials to comprehensively assess satisfaction with GRS.

## Conclusion

Our study stands as one of the initial reports to assess the outcomes of surgical interventions in transgender individuals in Iran. Additionally, it adds value to the limited body of literature by employing the WHOQOL-BRIEF instrument both before and after GRS. The results demonstrate that GRS brings about improvements across all aspects of QoL. However, this enhancement is less pronounced among patients from low socioeconomic backgrounds and rural areas. Therefore, an increase in targeted support and resources for individuals from these demographics is warranted.

## Data Availability

The datasets used and analyzed during the current study are available from the corresponding author on reasonable request and with permission of the Research Ethics Committee of the Shiraz University of Medical Sciences.

## References

[1] World Health Organization. International Classification of Diseases-ICD. 2009.

[2] Association AP. Diagnostic and statistical manual of mental disorders (DSM-5^®^): Am. J. Psychiatr. Pub; 2013. 10.1590/s2317-17822013000200017 24413388

[3] Judge C, O’Donovan C, Callaghan G, Gaoatswe G, O’Shea D. Gender dysphoria–prevalence and co-morbidities in an Irish adult population. Front Endocrinol. 2014;5:87. 10.3389/fendo.2014.00087 PMC4056308 24982651

[4] Chen M, Fuqua J, Eugster EA. Characteristics of referrals for gender dysphoria over a 13-year period. J Adolesc Health. 2016;58(3):369–71. 26903434 10.1016/j.jadohealth.2015.11.010 PMC5344189

[5] Arcelus J, Bouman WP, Van Den Noortgate W, Claes L, Witcomb G, Fernandez-Aranda F. Systematic review and meta-analysis of prevalence studies in transsexualism. Eur Psychiatry. 2015;30(6):807–15. 26021270 10.1016/j.eurpsy.2015.04.005

[6] Gilbert DA, Winslow BH, Gilbert DM, Jordan GH, Horton CE. Transsexual surgery in the genetic female. Clin Plast Surg. 1988;15(3):471–87. 3292116 10.1016/S0094-1298(20)31425-5

[7] Oort FJ. Using structural equation modeling to detect response shifts and true change. Qual Life Res. 2005;14(3):587–98. 16022054 10.1007/s11136-004-0830-y

[8] Oort FJ, Visser MR, Sprangers MA. An application of structural equation modeling to detect response shifts and true change in quality of life data from cancer patients undergoing invasive surgery. Qual Life Res. 2005;14(3):599–609. 16022055 10.1007/s11136-004-0831-x

[9] Shahriarirad R, Erfani A, Ranjbar K, Bazrafshan A, Mirahmadizadeh A. The mental health impact of COVID-19 outbreak: a Nationwide Survey in Iran. Int J Ment Health Syst. 2021;15(1):19. 33640006 10.1186/s13033-021-00445-3 PMC7913044

[10] Keramati MR, Yazd SMM, Omidi M, Keshvari A, Shahriarirad S, Shahriarirad R, et al. Translation, cross-cultural adaptation, and psychometric evaluation of the Persian (Farsi) version of the QoLAF (quality of life in patients with anal fistula) questionnaire. PLoS ONE. 2023;18(4):e0277170. 37027362 10.1371/journal.pone.0277170 PMC10081801

[11] Cohen-Kettenis PT, Gooren LJ. Transsexualism: a review of etiology, diagnosis and treatment. J Psychosom Res. 1999;46(4):315–33. 10340231 10.1016/S0022-3999(98)00085-3

[12] Gooren LJ, Giltay EJ, Bunck MC. Long-term treatment of transsexuals with cross-sex hormones: extensive personal experience. J Clin Endocrinol Metab. 2008;93(1):19–25. 17986639 10.1210/jc.2007-1809

[13] Johansson A, Sundbom E, Hojerback T, Bodlund O. A five-year follow-up study of Swedish adults with gender identity disorder. Arch Sex Behav. 2010;39(6):1429–37. 19816764 10.1007/s10508-009-9551-1

[14] De Cuypere G, Elaut E, Heylens G, Van Maele G, Selvaggi G, T’Sjoen G, et al. Long-term follow-up: psychosocial outcome of Belgian transsexuals after sex reassignment surgery. Sexologies. 2006;15(2):126–33. 10.1016/j.sexol.2006.04.002

[15] Wolfradt U, Neumann K. Depersonalization, self-esteem and body image in male-to-female transsexuals compared to male and female controls. Arch Sex Behav. 2001;30(3):301–10. 11330119 10.1023/A:1002752214526

[16] Fraser L. Depth psychotherapy with transgender people. Sex Relatsh Ther. 2009;24(2):126–42. 10.1080/14681990903003878

[17] Kuhn A, Bodmer C, Stadlmayr W, Kuhn P, Mueller MD, Birkhauser M. Quality of life 15 years after sex reassignment surgery for transsexualism. Fertil Steril. 2009;92(5):1685–9. e3. 18990387 10.1016/j.fertnstert.2008.08.126

[18] Davey A, Bouman WP, Arcelus J, Meyer C. Social support and psychological well-being in gender dysphoria: a comparison of patients with matched controls. J Sex Med. 2014;11(12):2976–85. 25155247 10.1111/jsm.12681

[19] Newfield E, Hart S, Dibble S, Kohler L. Female-to-male transgender quality of life. Qual Life Res. 2006;15(9):1447–57. 16758113 10.1007/s11136-006-0002-3

[20] Wierckx K, Van Caenegem E, Elaut E, Dedecker D, Van de Peer F, Toye K, et al. Quality of life and sexual health after sex reassignment surgery in transsexual men. J Sex Med. 2011;8(12):3379–88. 21699661 10.1111/j.1743-6109.2011.02348.x

[21] Weyers S, Elaut E, De Sutter P, Gerris J, T’Sjoen G, Heylens G, et al. Long-term assessment of the physical, mental, and sexual health among transsexual women. J Sex Med. 2009;6(3):752–60. 19040622 10.1111/j.1743-6109.2008.01082.x

[22] Castellano E, Crespi C, Dell’Aquila C, Rosato R, Catalano C, Mineccia V, et al. Quality of life and hormones after sex reassignment surgery. J Endocrinol Invest. 2015;38(12):1373–81. 26486135 10.1007/s40618-015-0398-0

[23] Bartolucci C, Gómez-Gil E, Salamero M, Esteva I, Guillamón A, Zubiaurre L, et al. Sexual quality of life in gender‐dysphoric adults before genital sex reassignment surgery. J Sex Med. 2015;12(1):180–8. 25401972 10.1111/jsm.12758

[24] Ahmadzad-Asl M, Jalali A-H, Alavi K, Naserbakht M, Taban M, Mohseninia-Omrani K, et al. The epidemiology of transsexualism in Iran. J Gay Lesbian Ment Health. 2010;15(1):83–93. 10.1080/19359705.2011.530580

[25] Talaei A, Hedjazi A, Badieyan Moosavi N, Dadgarmoghaddam M, Lotfinejad N, Khorashad BS. The epidemiology of gender dysphoria in Iran: the first nationwide study. Arch Sex Behav. 2022;51(4):1881–9. 35511409 10.1007/s10508-021-02250-y

[26] First MB. DSM-5^®^ handbook of differential diagnosis. American Psychiatric Pub; 2013.

[27] Nejat S, Montazeri A, Holakouie Naieni K, Mohammad K, Majdzadeh S. The World Health Organization quality of life (WHOQOL-BREF) questionnaire: translation and validation study of the Iranian version. J School Public Health Inst Public Health Res. 2006;4(4):1–12.

[28] World Health Organization. WHOQOL: Measuring Quality of Life [cited 2024 March]. https://www.who.int/tools/whoqol/whoqol-bref

[29] Skevington SM, Tucker C. Designing response scales for cross-cultural use in health care: data from the development of the UK WHOQOL. Br J Med Psychol. 1999;72(Pt 1):51–61. 10194572 10.1348/000711299159817

[30] Harper A, Power M. WHOQOL user manual. World Health Organization Edinburgh; 1999.

[31] Likert R. A technique for the measurement of attitudes. Arch Psychol. 1932;140:44–53.

[32] Cardoso da Silva D, Schwarz K, Fontanari AMV, Costa AB, Massuda R, Henriques AA, et al. WHOQOL-100 before and after sex reassignment surgery in Brazilian male-to-female transsexual individuals. J Sex Med. 2016;13(6):988–93. 27117529 10.1016/j.jsxm.2016.03.370

[33] Eftekhar Ardebili M, Janani L, Khazaei Z, Moradi Y, Baradaran HR. Quality of life in people with transsexuality after surgery: a systematic review and meta-analysis. Health Qual Life Outcomes. 2020;18(1):264. 32746856 10.1186/s12955-020-01510-0 PMC7397654

[34] Michel A, Ansseau M, Legros J-J, Pitchot W, Mormont C. The transsexual: what about the future? Eur Psychiatry. 2002;17(6):353–62. 12457746 10.1016/S0924-9338(02)00703-4

[35] Nuttbrock L, Hwahng S, Bockting W, Rosenblum A, Mason M, Macri M, et al. Psychiatric impact of gender-related abuse across the life course of male-to-female transgender persons. J Sex Res. 2010;47(1):12–23. 19568976 10.1080/00224490903062258

[36] Rezaei M, A Vahedian A. A. Comparison of depression, anxiety, stress and quality of life in dormitories students of Tarbiat Modares University. 2007.

[37] Blanchard R. Typology of male-to-female transsexualism. Arch Sex Behav. 1985;14(3):247–61. 4004548 10.1007/BF01542107

[38] Rakic Z, Starcevic V, Maric J, Kelin K. The outcome of sex reassignment surgery in Belgrade: 32 patients of both sexes. Arch Sex Behav. 1996;25:515–25. 8899143 10.1007/BF02437545

